# Compliance With England’s Calorie Labeling Regulations 3 Years After Policy Implementation

**DOI:** 10.1177/00333549251412799

**Published:** 2026-01-30

**Authors:** Oliver Huse, Alexandra Kalbus

**Affiliations:** 1 Population Health Innovation Lab, Department of Public Health, Environments and Society, London School of Hygiene & Tropical Medicine, London, UK

**Keywords:** food policy, calorie labeling, policy evaluation, nutrition, food environment

## Abstract

We examined compliance with England’s calorie labeling regulations, which require large out-of-home food businesses to display the calories of prepared food and drink at the point of choice, such as menus. By using website data from large out-of-home food businesses, we found that all businesses (n = 77) provided calorie labeling somewhere on their websites. However, fewer than half (48%; n = 37) did not provide calorie labels on the default menu (ie, the first menu that a consumer is likely to see). Compliance with the policy’s implementation guidance was greatest for the label’s position (81%; n = 62) and lowest for prominent formatting (40%; n = 31), while 71% (n = 55) of businesses provided the statement of daily calorie needs. We observed differences among types of out-of-home food businesses, but we did not test them because of the small sample size. Our results suggest imperfect adherence to England’s calorie labeling regulations, thus undermining the policy’s impact. As the policy’s review approaches, policy makers should consider strategies for ensuring compliance.

Diets high in energy-dense and nutrient-poor foods are a key contributor to poor health globally.^1^ In the United Kingdom, food prepared away from home contributes on average 300 kilocalories (kcal) per person per day.^2^ Meals served in large UK restaurants and fast-food chains are particularly calorie dense, reaching almost 1000 kcal per serving on average.^3^

To help consumers make informed choices when purchasing food prepared out of home, mandatory calorie labeling was introduced in England on April 6, 2022.^4^ All businesses with ≥250 employees are required to display the calorie content of food and nonalcoholic drink items prepared for immediate consumption at the point of choice, including menu boards, electronic menus, online menus, and third-party delivery app menus.^4^ The calorie label must correspond to the serving size offered, be displayed in kcal, and be accompanied by the statement “adults need around 2000 kcal a day,” referred to as a statement of daily calorie needs.^4^ The inclusion of menu labeling online is important, given the growth in availability and use of online food delivery platforms across the United Kingdom.^5^

Evidence suggests that the calorie labeling regulations have had minimal impact on consumer purchasing in England.^6,7^ One possible reason for this minimal effect is a low level of compliance by out-of-home food businesses: most businesses only partially comply with regulations,^8^ and enforcement mechanisms are lacking.^9^ In the United States, where mandatory calorie labeling was introduced in May 2018, a study published in 2020 showed that 186 of the 197 highest-grossing restaurant chains (94%) complied with regulations as of December 2018.^10^ Only 1 study^8^ has assessed compliance with England’s calorie labeling regulations; that study considered only in-store retail businesses a few months after policy implementation. The aim of this study was to assess compliance with calorie labeling regulations among large out-of-home food businesses in England 3 years after policy implementation and to assess whether compliance differed by type of out-of-home food business.

## Methods

We investigated out-of-home food businesses in alignment with those investigated by MenuTracker, a longitudinal database of online menus.^11^ MenuTracker exclusively includes businesses that have ≥250 employees and are therefore subject to labeling requirements. We included out-of-home food businesses if their websites provided either an integrated or downloadable menu that consumers might view to make a food purchasing decision. We excluded businesses that were aimed at business-to-business retail or did not present a food menu online.

Per the London School of Hygiene & Tropical Medicine’s good research practice policy, this study was exempt from ethical review because it did not include data from human or animal participants and all data collected were fully in the public domain.

One of 2 authors visited the websites of all out-of-home food businesses during April 1-4, 2025, from the same location. Both authors collected data for a subset of 20 websites to assess interrater reliability, which was 100% for the default menu; 90% for eligible items, label position, and reference statement; and 85% for prominent formatting. We visited websites in private browser mode, and we accessed the online food menu via the most direct pathway to assess compliance with England’s calorie labeling regulations (eTable in the Supplement).^4^ We chose compliance characteristics for assessment based on the calorie labeling guidance and feasibility of assessment. All food and drink items on a menu were eligible for assessment, except alcoholic beverages, unpackaged or “loose” produce (eg, loose bananas), and temporary or seasonal menu items.^4^ If a website required the visitor’s location, we provided a standardized postcode (WC1H 9SH), and we selected the closest available outlet. As a sensitivity analysis to assess compliance with regulations across business locations, we visited the websites of a random sample of out-of-home food businesses and entered a different postcode (NE1 5DL).

We conducted a descriptive data analysis in R version 4.5.0 (R Core Team). We did not test differences among types of out-of-home food businesses because of the small sample size.

## Results

**Figure 1.**
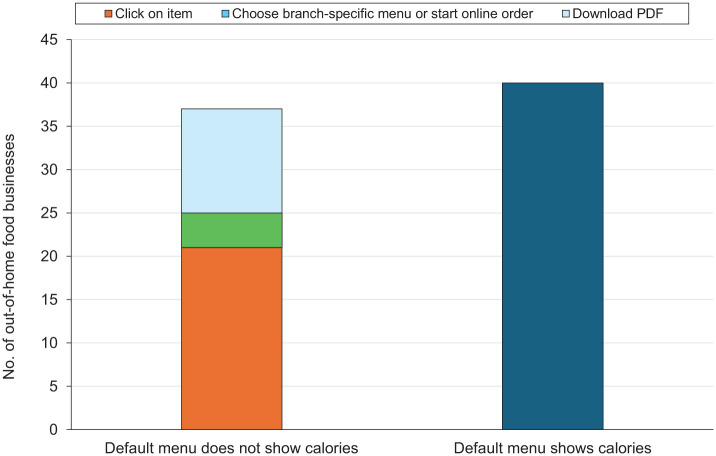
Number of out-of-home food business that show (vs do not show) calorie information on their website’s default menu, per implementation guidance of England’s calorie labeling regulations,^4^ and the steps required to view calorie information if not shown on the default menu, United Kingdom, April 2025. “Default menu” refers to the first menu that a consumer is likely to see when visiting the website of a food business. Data source: analysis of data from the websites of 77 large out-of-home food businesses in the United Kingdom.

We included 77 out-of-home food businesses in this study: 16 cafes and bakeries; 20 pubs, bars, and inns; 17 restaurants; 23 fast-food and takeaway outlets; and 1 sport and entertainment venue. The sport and entertainment venue was excluded where data were stratified by business type. All 77 businesses displayed calorie labeling on at least 1 menu on their websites. A little more than half (52%; n = 40) showed calorie labeling on their default menus (Figure 1). Of those that did not (n = 37), 57% (n =21) required customers to click on the menu item, 32% (n = 12) required labeled menus to be downloaded as PDFs through either a click (n = 11) or a QR code (quick response; n = 1), and 11% (n = 4) required customers to choose a branch-specific menu or start an online order.

**Figure 2.**
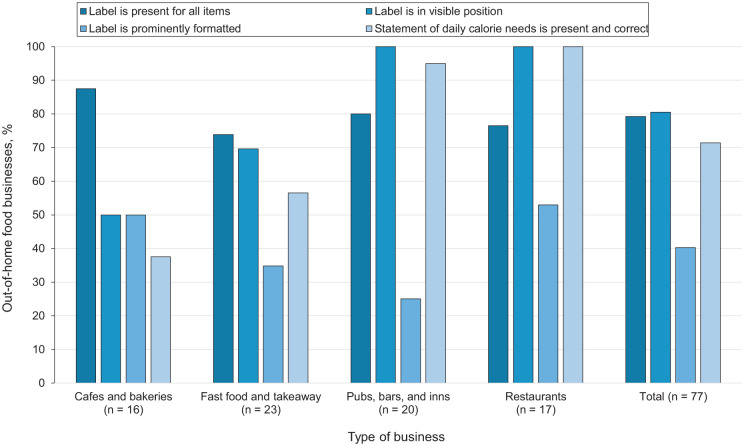
Percentage of out-of-home food businesses (N = 77) that complied with 4 characteristics of calorie labeling on menus, by food business type, United Kingdom, April 2025. Compliance was assessed according to implementation guidance of England’s calorie labeling regulations.^4^ The 1 sport and entertainment venue is included in the total but not shown separately. Data source: analysis of data from the websites of 77 large out-of-home food businesses in the United Kingdom.

Calorie labels were displayed for all eligible items by 79% (n = 61) of out-of-home food businesses (Figure 2). Compliance was greatest for the calorie label’s position (close to the item’s name, price, or description), with 81% (n = 62) of businesses compliant. However, only 40% (n = 31) of food outlets presented calorie labeling in at least as prominent font and size as the item name, price, or description. The statement of daily calorie needs was present and correct on the menus of 71% (n = 55) of the food businesses. Overall, 20% (n = 15) of businesses were compliant with all assessed criteria, of which only 1 was a fast-food outlet.

The sensitivity analysis that compared the labeling compliance characteristics of out-of-home food businesses in a different location for a subset of the included businesses found no differences in compliance.

## Discussion

Although all 77 out-of-home food outlets that we studied provided calorie information on their websites, many used tactics to avoid displaying calories to the consumer at the point of choice. Fewer than half of the 77 food businesses did not display calorie labeling on the default menu (ie, the first point of choice) but instead required additional steps to view calorie-labeled menus. Calorie labels were frequently displayed in a less prominent font style, size, or color as compared with other menu text. Likewise, several websites used alternative wording for the statement of daily calorie needs or did not display this statement at all. These omissions and alternative choices are all in violation of what the calorie labeling regulations in England require businesses to do.^4^ While we cannot determine whether these violations are due to intentional actions by out-of-home food businesses or errors in interpretation of the regulations, the end result is the same—the undermining of the policy’s impact.

Polden et al reported that several months after policy implementation in England, 80% and 67% of out-of-home food businesses provided calorie labeling on any and all menus available in-store, respectively, while only 6% displayed prominently formatted calorie labeling and 45% presented a clear and prominent statement of daily calorie needs.^8^ Our study builds on this work by assessing the menus encountered by consumers online 3 years after policy implementation and examining the default menu. Considering the reported lack of enforcement from local government,^9^ we are not surprised that out-of-home food outlets are not consistently complying with England’s calorie labeling regulations.

To the best of our knowledge, this is 1 of just 2 studies to assess compliance with the regulations; however, several limitations apply. First, while unlikely, online menus may not represent in-store menus. Second, we cannot comment on the entire out-of-home food sector because our study included only businesses listed in the MenuTracker database. These are large businesses that are subject to calorie labeling, provided nutritional information in 2021, and are estimated to constitute 25% of businesses in the out-of-home food sector.^11^ Third, our analysis was descriptive and cross-sectional, meaning that we could not assess shifts in compliance over time and findings should be interpreted cautiously, although they reflect the existing literature on this topic.^8^

Our results suggest that out-of-home food outlets are inconsistently adhering to England’s calorie labeling regulations, thus undermining the policy’s impact. As the review of the policy approaches,^4^ it will be important for the UK government to consider (1) how to ensure compliance and (2) what other strategies—such as alternative labeling options, fiscal approaches, and marketing restrictions—best support consumers in achieving a healthy diet in out-of-home food settings.

## Supplemental Material

sj-docx-1-phr-10.1177_00333549251412799 – Supplemental material for Compliance With England’s Calorie Labeling Regulations 3 Years After Policy Implementation Supplemental material, sj-docx-1-phr-10.1177_00333549251412799 for Compliance With England’s Calorie Labeling Regulations 3 Years After Policy Implementation by Oliver Huse and Alexandra Kalbus in Public Health Reports®

sj-docx-2-phr-10.1177_00333549251412799 – Supplemental material for Compliance With England’s Calorie Labeling Regulations 3 Years After Policy Implementation Supplemental material, sj-docx-2-phr-10.1177_00333549251412799 for Compliance With England’s Calorie Labeling Regulations 3 Years After Policy Implementation by Oliver Huse and Alexandra Kalbus in Public Health Reports®

sj-pdf-4-phr-10.1177_00333549251412799 – Supplemental material for Compliance With England’s Calorie Labeling Regulations 3 Years After Policy Implementation Supplemental material, sj-pdf-4-phr-10.1177_00333549251412799 for Compliance With England’s Calorie Labeling Regulations 3 Years After Policy Implementation by Oliver Huse and Alexandra Kalbus in Public Health Reports®

sj-pdf-5-phr-10.1177_00333549251412799 – Supplemental material for Compliance With England’s Calorie Labeling Regulations 3 Years After Policy Implementation Supplemental material, sj-pdf-5-phr-10.1177_00333549251412799 for Compliance With England’s Calorie Labeling Regulations 3 Years After Policy Implementation by Oliver Huse and Alexandra Kalbus in Public Health Reports®

sj-xlsx-3-phr-10.1177_00333549251412799 – Supplemental material for Compliance With England’s Calorie Labeling Regulations 3 Years After Policy Implementation Supplemental material, sj-xlsx-3-phr-10.1177_00333549251412799 for Compliance With England’s Calorie Labeling Regulations 3 Years After Policy Implementation by Oliver Huse and Alexandra Kalbus in Public Health Reports®
