# Supplementary material for: Compliance With England’s Calorie Labeling Regulations 3 Years After Policy Implementation
Source: Public Health Rep. 2026 Jan 30:00333549251412799. Online ahead of print. doi: 10.1177/00333549251412799 (PMC12861396; doi:10.1177/00333549251412799)
Supplement: sj-docx-1-phr-10.1177_00333549251412799 – Supplemental material for Compliance With England’s Calorie Labeling Regulations 3 Years After Policy Implementation [file sj-docx-1-phr-10.1177_00333549251412799.docx]

############# Calorie labelling compliance analysis ################

# load packages

library(dplyr) # data manipulation (incl %>% pipe)

library(ggplot2) # for plots

library(here) # relative folder location - no need to type full file paths if script in same folder as data/other outputs

library(readxl) # read in excel files

library(viridis) # for colour palette

library(scales) # for the percent stacked bar chart (to print % on y-axis)

# load data

d <- read_excel(here("Calorie labelling compliance data.xlsx")) # we can use shorter file path here relative to where the the script is saved

# View data

View(d)

## Data prep ####

# drop 'notes' row and variables not needed in this analysis, and shorten variable names

d <- d %>%

slice(-1) %>% # removes the first row (notes)

select(`Restaurant name`, Type, `Labelling provided anywhere`, `Default menu shows calories`, `Steps to see labelled menu`, `Labelled menu more than 1 click away`, `Labelled menu more than 2 clicks away`, `Labels present for all items`, `Label position`, `Label prominence`, `Reference statement`, `Labels shown when ordering online`) %>%

rename(`Statement of daily calorie needs` = `Reference statement`)

count(d) # 77 outlets

table(d$`Labelling provided anywhere`)

# we grouped Western & Asian Fast Food (n=3) together

# for stratification by business type, we excluded the 1 sport 7 entertainment venue (n=76)

d_type <- d %>%

filter(Type != "Sport & Entertainment") %>%

mutate(Type = recode(Type,

"Asian Fast Food" = "Fast Food & Takeaway",

"Cafes and Bakeries" = "Cafes & Bakeries",

"Pubs, Bars & Inns" = "Pubs, Bars & Inns",

"Restaurants" = "Restaurants",

"Western Fast Food & Takeaway" = "Fast Food & Takeaway"))

## Summary stats ####

d_type %>% group_by(Type) %>% count() # plus 1 sport & entertainment venue

### Does the default menu display calories? ####

d %>%

group_by(`Default menu shows calories`) %>%

summarise(`Number of restaurants` = n()) %>%

mutate(`%` = `Number of restaurants` / sum(`Number of restaurants`) * 100)

# by restaurant type type

d_type %>%

group_by(`Type`, `Default menu shows calories`) %>%

summarise(`Number of restaurants` = n()) %>%

mutate(`% default menu shows kcal` = `Number of restaurants` / sum(`Number of restaurants`) * 100) %>%

filter(`Default menu shows calories` == 'Yes') %>%

select(Type, `% default menu shows kcal`)

# chi-square test by business type (not reported due to small sample size)

#contingency_table <- table(d_type$Type, d_type$`Default menu shows calories`)

#contingency_table # same table as above - chi square command needs a table as input

#chisq.test(contingency_table)

### Where the default does not show calories, what are the necessary steps to see calorie labels (n=37)? ####

d %>%

filter(`Default menu shows calories`=='No') %>%

group_by(`Steps to see labelled menu`) %>%

summarise(`Number of restaurants` = n()) %>%

mutate(`%` = `Number of restaurants` / sum(`Number of restaurants`) * 100)

# by business type

d_type %>%

filter(`Default menu shows calories`=='No') %>%

group_by(`Type`,`Steps to see labelled menu`) %>%

summarise(`Number of restaurants` = n()) %>%

mutate(`%` = `Number of restaurants` / sum(`Number of restaurants`) * 100)

### How many clicks to get to labelled menu where this is not the default? ####

# Is the labelled menu more than 1 click away?

d %>%

filter(`Default menu shows calories`=='No') %>%

group_by(`Labelled menu more than 1 click away`) %>%

summarise(`Number of restaurants` = n()) %>%

mutate(`%` = `Number of restaurants` / sum(`Number of restaurants`) * 100)

# Is the labelled menu more than 2 clicks away?

d %>%

filter(`Default menu shows calories`=='No') %>%

group_by(`Labelled menu more than 2 clicks away`) %>%

summarise(`Number of restaurants` = n()) %>%

mutate(`%` = `Number of restaurants` / sum(`Number of restaurants`) * 100)

### Is labelling available for all eligble items? ####

d %>%

group_by(`Labels present for all items`) %>%

summarise(`Number of restaurants` = n()) %>%

mutate(`%` = `Number of restaurants` / sum(`Number of restaurants`) * 100)

# by business type

d_type %>%

group_by(`Type`, `Labels present for all items`) %>%

summarise(`Number of restaurants` = n()) %>%

mutate(`% compliant` = `Number of restaurants` / sum(`Number of restaurants`) * 100) %>%

filter(`Labels present for all items` == 'Yes') %>%

select(Type, `% compliant`)

# save % for compliance graph

c_items <- d_type %>%

group_by(`Type`, `Labels present for all items`) %>%

summarise(`Number of restaurants` = n()) %>%

mutate(`%` = `Number of restaurants` / sum(`Number of restaurants`) * 100) %>%

filter(`Labels present for all items` == 'Yes') %>%

select(Type, `%`) %>%

mutate(compliance = "Label is present for all items")

all_c <- d %>%

group_by(`Labels present for all items`) %>%

summarise(`Number of restaurants` = n()) %>%

mutate(`%` = `Number of restaurants` / sum(`Number of restaurants`) * 100,

Type = "Total",

compliance = "Label is present for all items") %>%

filter(`Labels present for all items` == 'Yes') %>%

select(Type, `%`, compliance)

### How visible is the calorie label (position)? ####

d %>%

group_by(`Label position`) %>%

summarise(`Number of restaurants` = n()) %>%

mutate(`%` = `Number of restaurants` / sum(`Number of restaurants`) * 100)

# by business type

d_type %>%

group_by(`Type`, `Label position`) %>%

summarise(`Number of restaurants` = n()) %>%

mutate(`% compliant` = `Number of restaurants` / sum(`Number of restaurants`) * 100) %>%

filter(`Label position` == 'Close to name, price or description') %>%

select(Type, `% compliant`)

# save % for compliance graph

c_position <- d_type %>%

group_by(`Type`, `Label position`) %>%

summarise(`Number of restaurants` = n()) %>%

mutate(`%` = `Number of restaurants` / sum(`Number of restaurants`) * 100) %>%

filter(`Label position` == 'Close to name, price or description') %>%

select(Type, `%`) %>%

mutate(compliance = "Label is in visible position")

all_c <- d %>%

group_by(`Label position`) %>%

summarise(`Number of restaurants` = n()) %>%

mutate(`%` = `Number of restaurants` / sum(`Number of restaurants`) * 100,

Type = "Total",

compliance = "Label is in visible position") %>%

filter(`Label position` == 'Close to name, price or description') %>%

select(Type, `%`, compliance) %>% rbind(all_c)

### How prominently formatted is the label? ####

d %>%

group_by(`Label prominence`) %>%

summarise(`Number of restaurants` = n()) %>%

mutate(`% compliant` = `Number of restaurants` / sum(`Number of restaurants`) * 100)

# by business type

d_type %>%

group_by(`Type`, `Label prominence`) %>%

summarise(`Number of restaurants` = n()) %>%

mutate(`% compliant` = `Number of restaurants` / sum(`Number of restaurants`) * 100) %>%

filter(`Label prominence` == 'Yes') %>%

select(Type, `% compliant`)

# save % for compliance graph

c_prominence <- d_type %>%

group_by(`Type`, `Label prominence`) %>%

summarise(`Number of restaurants` = n()) %>%

mutate(`%` = `Number of restaurants` / sum(`Number of restaurants`) * 100) %>%

filter(`Label prominence` == 'Yes') %>%

select(Type, `%`) %>%

mutate(compliance = "Label is prominently formatted")

all_c <- d %>%

group_by(`Label prominence`) %>%

summarise(`Number of restaurants` = n()) %>%

mutate(`%` = `Number of restaurants` / sum(`Number of restaurants`) * 100,

Type = "Total",

compliance = "Label is prominently formatted") %>%

filter(`Label prominence` == 'Yes') %>%

select(Type, `%`, compliance) %>% rbind(all_c)

### Statement of daily calorie needs ####

d %>%

mutate(`Statement of daily calorie needs compliant` = ifelse(`Statement of daily calorie needs`=="Statement is present and clear" | `Statement of daily calorie needs`=="Statement is in fineprint", "Yes", "No")) %>%

group_by(`Statement of daily calorie needs compliant`) %>%

summarise(`Number of restaurants` = n()) %>%

mutate(`%` = `Number of restaurants` / sum(`Number of restaurants`) * 100)

# by business type

d_type %>%

mutate(reference_compliant = ifelse(`Statement of daily calorie needs`=="Statement is present and clear" | `Statement of daily calorie needs`=="Statement is in fineprint", "Yes", "No")) %>%

group_by(`Type`, reference_compliant) %>%

summarise(`Number of restaurants` = n()) %>%

mutate(`% compliant` = `Number of restaurants` / sum(`Number of restaurants`) * 100) %>%

filter(reference_compliant == 'Yes') %>%

select(Type, `% compliant`)

# save % for compliance graph

c_reference <- d_type %>%

mutate(reference_compliant = ifelse(`Statement of daily calorie needs`=="Statement is present and clear" | `Statement of daily calorie needs`=="Statement is in fineprint", "Statement of daily calorie needs", "not compliant")) %>%

group_by(`Type`, reference_compliant) %>%

summarise(`Number of restaurants` = n()) %>%

mutate(`%` = `Number of restaurants` / sum(`Number of restaurants`) * 100) %>%

filter(reference_compliant == "Statement of daily calorie needs") %>%

select(Type, `%`) %>%

mutate(compliance = "Statement of daily calorie needs \nis present and correct")

all_c <- d %>%

mutate(reference_compliant = ifelse(`Statement of daily calorie needs`=="Statement is present and clear" | `Statement of daily calorie needs`=="Statement is in fineprint", "Statement of daily calorie needs", "not compliant")) %>%

group_by(reference_compliant) %>%

summarise(`Number of restaurants` = n()) %>%

mutate(`%` = `Number of restaurants` / sum(`Number of restaurants`) * 100,

Type = "Total",

compliance = "Statement of daily calorie needs \nis present and correct") %>%

filter(reference_compliant == "Statement of daily calorie needs") %>%

select(Type, `%`, compliance) %>% rbind(all_c)

### Compliance with all criteria ####

# default menu + all items labelled + label formatting & position + statement

d %>%

mutate(reference_compliant = ifelse(`Statement of daily calorie needs`=="Statement is present and clear" | `Statement of daily calorie needs`=="Statement is in fineprint", "Statement of daily calorie needs", "not compliant"),

`Compliant with all criteria` = ifelse(`Default menu shows calories` == "Yes" & `Labels present for all items` == "Yes" & `Label position` == "Close to name, price or description" & `Label prominence`== "Yes" & reference_compliant == "Statement of daily calorie needs", "Yes", "No")) %>%

group_by(`Compliant with all criteria`) %>%

summarise(`Number of restaurants` = n()) %>%

mutate(`% compliant with all criteria` = `Number of restaurants` / sum(`Number of restaurants`) * 100) %>%

filter(`Compliant with all criteria` == 'Yes') %>%

select(`Number of restaurants`,`% compliant with all criteria`)

# By business type

d_type <- d_type %>%

mutate(reference_compliant = ifelse(`Statement of daily calorie needs`=="Statement is present and clear" | `Statement of daily calorie needs`=="Statement is in fineprint", "Statement of daily calorie needs", "not compliant"),

`Compliant with all criteria` = ifelse(`Default menu shows calories` == "Yes" & `Labels present for all items` == "Yes" & `Label position` == "Close to name, price or description" & `Label prominence`== "Yes" & reference_compliant == "Statement of daily calorie needs", "Yes", "No"))

d_type %>%

group_by(Type,`Compliant with all criteria`) %>%

summarise(`Number of restaurants` = n()) %>%

mutate(`% compliant with all criteria` = `Number of restaurants` / sum(`Number of restaurants`) * 100) %>%

filter(`Compliant with all criteria` == 'Yes') %>%

select(Type,`Number of restaurants`,`% compliant with all criteria`)

# chi-square test by business type (not reported due to small sample size)

#contingency_table <- table(d_type$Type, d_type$`Compliant with all criteria`)

#contingency_table # same table as above - chi square command needs a table as input

#chisq.test(contingency_table)

### Ordering online ####

d %>%

group_by(`Labels shown when ordering online`) %>%

summarise(`Number of restaurants` = n()) %>%

mutate(`%` = `Number of restaurants` / sum(`Number of restaurants`) * 100)

# by business type

d_type %>%

group_by(`Type`, `Labels shown when ordering online`) %>%

summarise(`Number of restaurants` = n()) %>%

mutate(`%` = `Number of restaurants` / sum(`Number of restaurants`) * 100)

## Graphs #####

### default menu with labelling & steps to get to it ####

# create data for plotting

d_graph <- d %>%

group_by(`Default menu shows calories`, `Steps to see labelled menu`) %>%

summarise(value = n()) %>%

mutate(`Default menu shows calories` = factor(`Default menu shows calories`, levels = c("Yes", "No")), # makes the 'yes' bar appear first

`Steps to see labelled menu` = factor(ifelse(is.na(`Steps to see labelled menu`), "Default menu is labelled", recode(`Steps to see labelled menu`,

"Click on item" = "Click on item",

"choose branch-specific menu or start online order" = "Choose branch-specific menu \nor start online order",

"download pdf" = "Download PDF",

"download pdf - with QR code" = "Download PDF")),

levels = c("Default menu is labelled", "Click on item", "Download PDF", "Choose branch-specific menu \nor start online order")))

# View the underlying numbers

d_graph

colours = viridis(4) # set graph colours

ggplot(d_graph, aes(fill=`Steps to see labelled menu`, y=value, x=`Default menu shows calories`)) + # tell R we want to make a figure (ggplot) with which data and variables to use

geom_bar(position="stack", stat="identity") + # specify what type of graph

scale_fill_manual(values = colours) + # specify colours (of 'fill' - here the steps to see the labelled menu); taken from the viridis palette

labs(y = "Number of food businesses") + # specify axis title

theme_classic() # set general formatting

#ggsave(here("Fig 1. Steps to see labelled menu.tiff"), width = 6, height=4) # saves the last produced figure into the specified file, in specified format and dimensions

### default menu showing calories by business type (not shown in report) ####

total_graph <- d %>%

mutate(Type = "Total") %>%

group_by(Type, `Default menu shows calories`) %>%

summarise(value = n()) %>%

mutate(pct= prop.table(value) * 100)

d_graph <- d_type %>%

group_by(Type, `Default menu shows calories`) %>%

summarise(value = n()) %>%

mutate(pct= prop.table(value) * 100) %>%

rbind(total_graph) %>%

mutate(Type = factor(Type, levels = c("Total", "Cafes & Bakeries", "Pubs, Bars & Inns", "Restaurants", "Fast Food & Takeaway")))

ggplot(d_graph, aes(x=Type, y=value, fill=`Default menu shows calories`)) +

geom_bar(position="fill", stat="identity") +

scale_fill_manual(values = c("#31688EFF", "#35B779FF")) +

labs(y = NULL, x = NULL) +

theme_classic() +

scale_y_continuous(labels = scales::percent)

#ggsave(here("Type and default menu percent stacked.tiff"), width = 8.5, height = 5)

### Labelling compliance graph ####

d_graph <- rbind(c_items, c_position, c_prominence, c_reference, all_c) %>% # row-binds datasets created above

mutate(Type = factor(Type, levels = c("Total", "Cafes & Bakeries", "Pubs, Bars & Inns", "Restaurants", "Fast Food & Takeaway")),

compliance = factor(compliance, levels = c("Label is present for all items", "Label is in visible position", "Label is prominently formatted", "Statement of daily calorie needs \nis present and correct"))) # the \n in the text makes the text break to the next line in figure

ggplot(d_graph, aes(x=Type, y=`%`/100, fill=compliance)) +

geom_bar(position="dodge", stat="identity") +

scale_fill_manual(values = viridis(5)) +

labs(y = "Compliant", x = NULL, fill = NULL) + # fill=NULL removes legend title

theme_classic() +

scale_y_continuous(labels = scales::percent)

#ggsave(here("Fig.2 Compliance characteristics by type.tiff"), width=9, height=5.5)
