# Supplementary material for: Compliance With England’s Calorie Labeling Regulations 3 Years After Policy Implementation
Source: Public Health Rep. 2026 Jan 30:00333549251412799. Online ahead of print. doi: 10.1177/00333549251412799 (PMC12861396; doi:10.1177/00333549251412799)
Supplement: sj-docx-2-phr-10.1177_00333549251412799 – Supplemental material for Compliance With England’s Calorie Labeling Regulations 3 Years After Policy Implementation [file sj-docx-2-phr-10.1177_00333549251412799.docx]

**eTable: England Calorie labelling guidance**

| **Regulation scope / Compliance characteristics** | **Guidance (1)** |
| --- | --- |
| Businesses in scope | “*The requirement to calorie label extends to any business with 250 or more employees which offers for sale non-prepacked food or drink suitable for immediate consumption by the person who buys it.*” |
| Foods and beverages in scope | “*Food which is:*   - *Offered for sale in a form which is suitable for immediate consumption* - *Not prepacked food* - *Not exempt food* [e.g. loose fresh fruits or vegetables]*.*” |
| Default menu offering | *The Regulations permit businesses to provide a menu without the required calorie information at the express request of the customer. While a menu without calorie information can be provided at the request of the customer, a menu with the required calorie information must be offered to the customer by default.* |
| Displaying calorie information | “*Businesses selling food in scope of the Regulations must:*   - *Display the energy content of the food in kilocalories (kcal)* - *Reference the size of the portion to which the calorie information relates* - *Display the statement that ‘adults need around 2000 kcal a day’.*” |
| Definition of ‘point of choice’ | “*Calorie information, the reference to portion size, and the statement of daily calorie needs must be displayed clearly and prominently at the ‘point of choice’ for the customer; this is considered as any place where customers choose what food to buy.*  …  [This includes] *food chosen from a menu (such as menu boards, electronic menus, online and third-party delivery app menus).*” |
| Label positioning | “*Energy content of food and drink must be calculated in kilocalories (kcal) and be displayed:*   - *where food is chosen from a menu: on the menu, next to the description or the price of the food.*” |
| Label formatting | *In all cases, the information businesses are required to display must be easily visible, clearly legible, and not in any way hidden or obscured by other written or pictorial matter, or any other intervening material. Ensuring the information is easily visible could be achieved by using the same font type or style of lettering, colour, size and background of the description or price of the corresponding food item.* |

1. UK Government. Calorie labelling in the out of home sector: implementation guidance. 2021.
