# Supplementary material for: Compliance With England’s Calorie Labeling Regulations 3 Years After Policy Implementation
Source: Public Health Rep. 2026 Jan 30:00333549251412799. Online ahead of print. doi: 10.1177/00333549251412799 (PMC12861396; doi:10.1177/00333549251412799)
Supplement: sj-pdf-4-phr-10.1177_00333549251412799 – Supplemental material for Compliance With England’s Calorie Labeling Regulations 3 Years After Policy Implementation [file sj-pdf-4-phr-10.1177_00333549251412799.pdf]

Data_for_Figure_2

	Percent of businesses	Compliance criterion	Total number of businesses
**Cafes & Bakeries**	87.5	Label is present for all items	16
**Cafes & Bakeries**	50	Label is in visible position	16
**Cafes & Bakeries**	50	Label is prominently formatted	16
**Cafes & Bakeries**	37.5	Reference statement is present and correct	16
**Fast Food & Takeaway**	73.91304347826090	Label is present for all items	23
**Fast Food & Takeaway**	69.56521739130430	Label is in visible position	23
**Fast Food & Takeaway**	34.78260869565220	Label is prominently formatted	23
**Fast Food & Takeaway**	56.52173913043480	Reference statement is present and correct	23
**Pubs, Bars & Inns**	80	Label is present for all items	20
**Pubs, Bars & Inns**	100	Label is in visible position	20
**Pubs, Bars & Inns**	25	Label is prominently formatted	20
**Pubs, Bars & Inns**	95	Reference statement is present and correct	20
**Restaurants**	76.47058823529410	Label is present for all items	17
**Restaurants**	100	Label is in visible position	17
**Restaurants**	52.94117647058820	Label is prominently formatted	17
**Restaurants**	100	Reference statement is present and correct	17
**Total**	71.42857142857140	Reference statement is present and correct	77
**Total**	40.25974025974030	Label is prominently formatted	77
**Type**	80.51948051948050	Label is in visible position	77
**Total**	79.22077922077920	Label is present for all items	77
