# Supplementary material for: Compliance With England’s Calorie Labeling Regulations 3 Years After Policy Implementation
Source: Public Health Rep. 2026 Jan 30:00333549251412799. Online ahead of print. doi: 10.1177/00333549251412799 (PMC12861396; doi:10.1177/00333549251412799)

Data_for_Figure_1

Default menu shows calories	Steps to see labelled menu	Number of businesses
**No**	Click on item	21
**No**	Choose branch-specific menu or start online order	4
**No**	Download PDF	11
**No**	Download PDF	1
**Yes**	Default menu is labelled	40

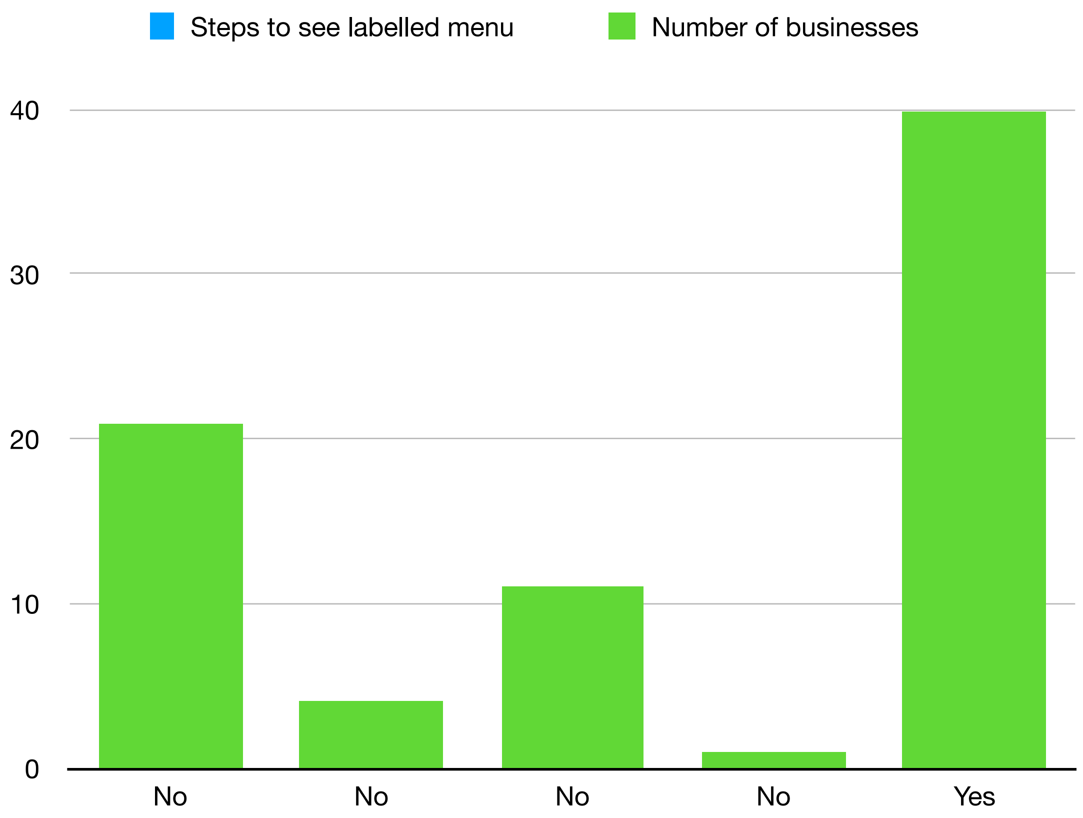

Supplement: sj-pdf-5-phr-10.1177_00333549251412799 – Supplemental material for Compliance With England’s Calorie Labeling Regulations 3 Years After Policy Implementation [file sj-pdf-5-phr-10.1177_00333549251412799.pdf]
